# Supplementary material for: Interneuron diversity in the human dorsal striatum
Source: Nat Commun. 2024 Jul 22;15:6164. doi: 10.1038/s41467-024-50414-w (PMC11263574; doi:10.1038/s41467-024-50414-w)
Supplement: Supplementary file 1 — Supplementary Information [file 41467_2024_50414_MOESM1_ESM.pdf]

Supplementary Figures

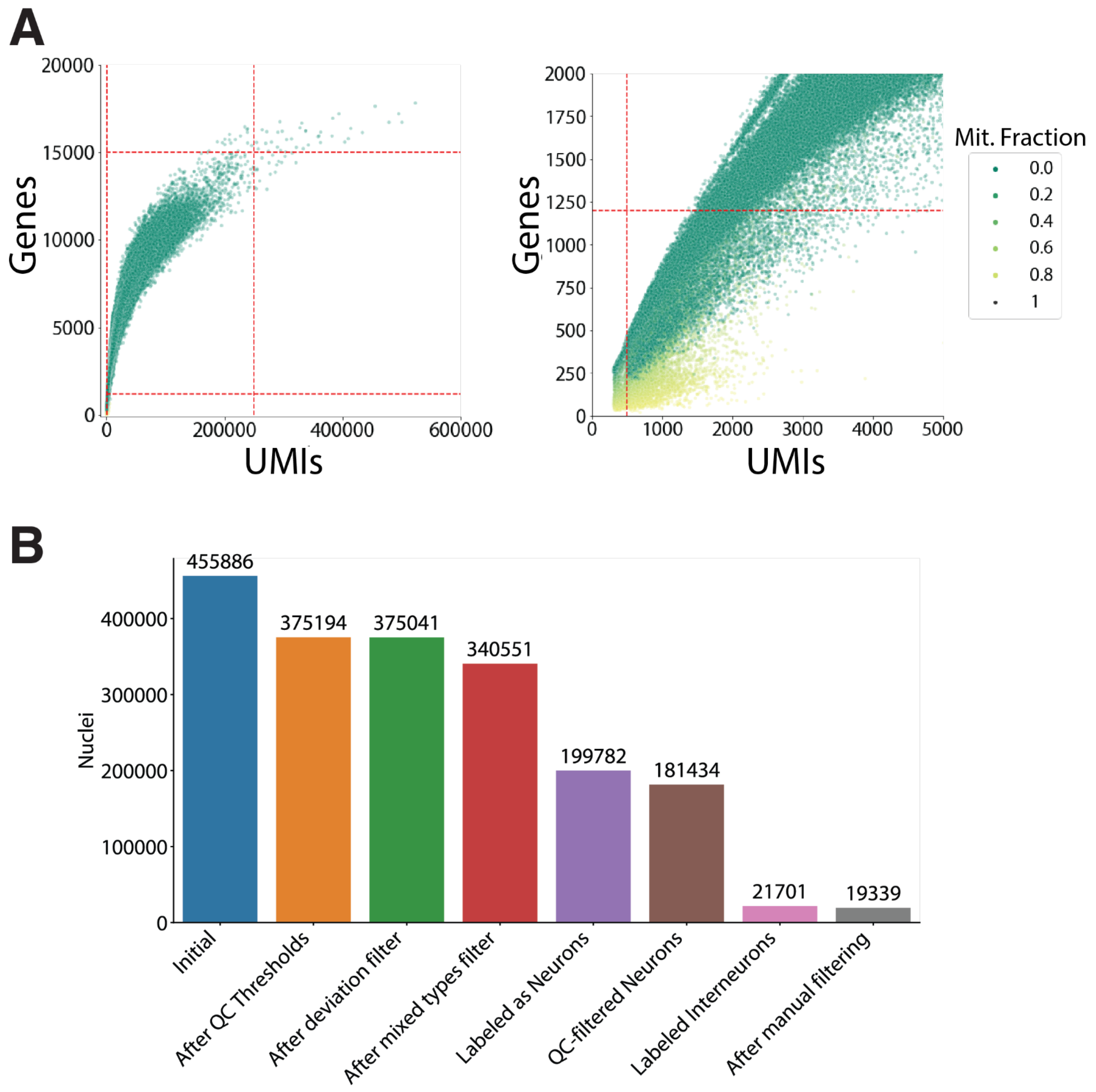

Supplementary Figure 1. Quality control metrics of the snRNA-seq data. A. Distribution of the number of genes and counts per nucleus, colored by the fraction of total detected UMIs corresponding to mitochondrial gene products. The values chosen for the initial thresholds are shown in red (see Methods). B. Number of nuclei retained at each step of the analysis.

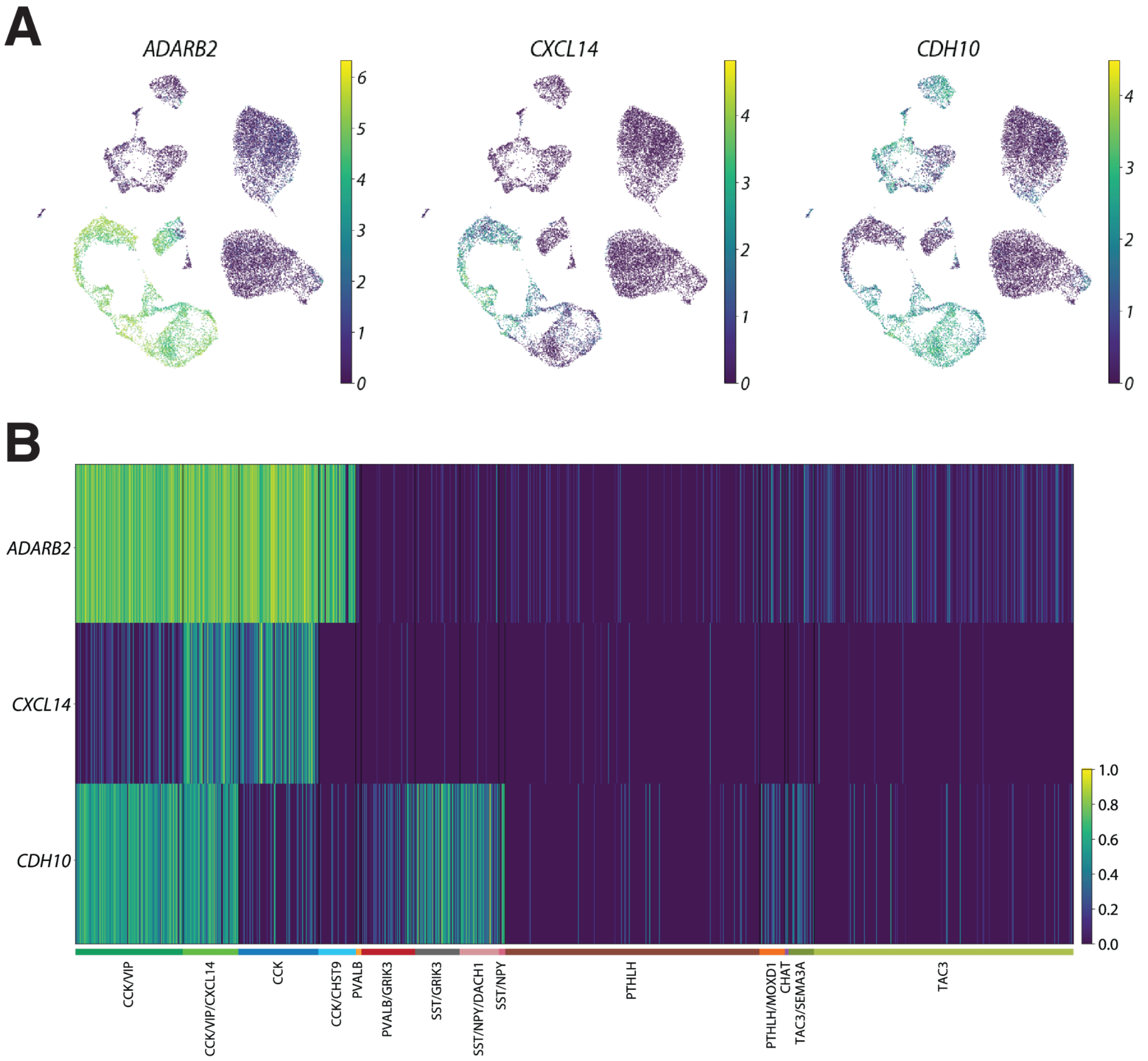

Supplementary Figure 2. Expression of *ADARB2*, *CXCL14*, and *CDH10* distinguishes subclasses of *CCK*-expressing interneurons. A. uniform manifold approximation projection (UMAP) of all the interneuron nuclei showing the normalized expression of *ADARB2*, *CXCL14*, and *CDH10*. B. Normalized expression of the same genes on the nuclei ordered by interneuron subclass. As the heatmap shows, *CXCL14* and *CDH10* could be used to distinguish the different subclasses of *CCK*+ interneurons that also co-express *ADARB2*: *CDH10*+/*CXCL14*- (CCK/VIP); *CDH10*+/*CXCL14*+ (CCK/VIP/CXCL14); *CDH10*-/*CXCL14*+ (CCK); *CDH10*-/*CXCL14*- (CCK/CHST9).

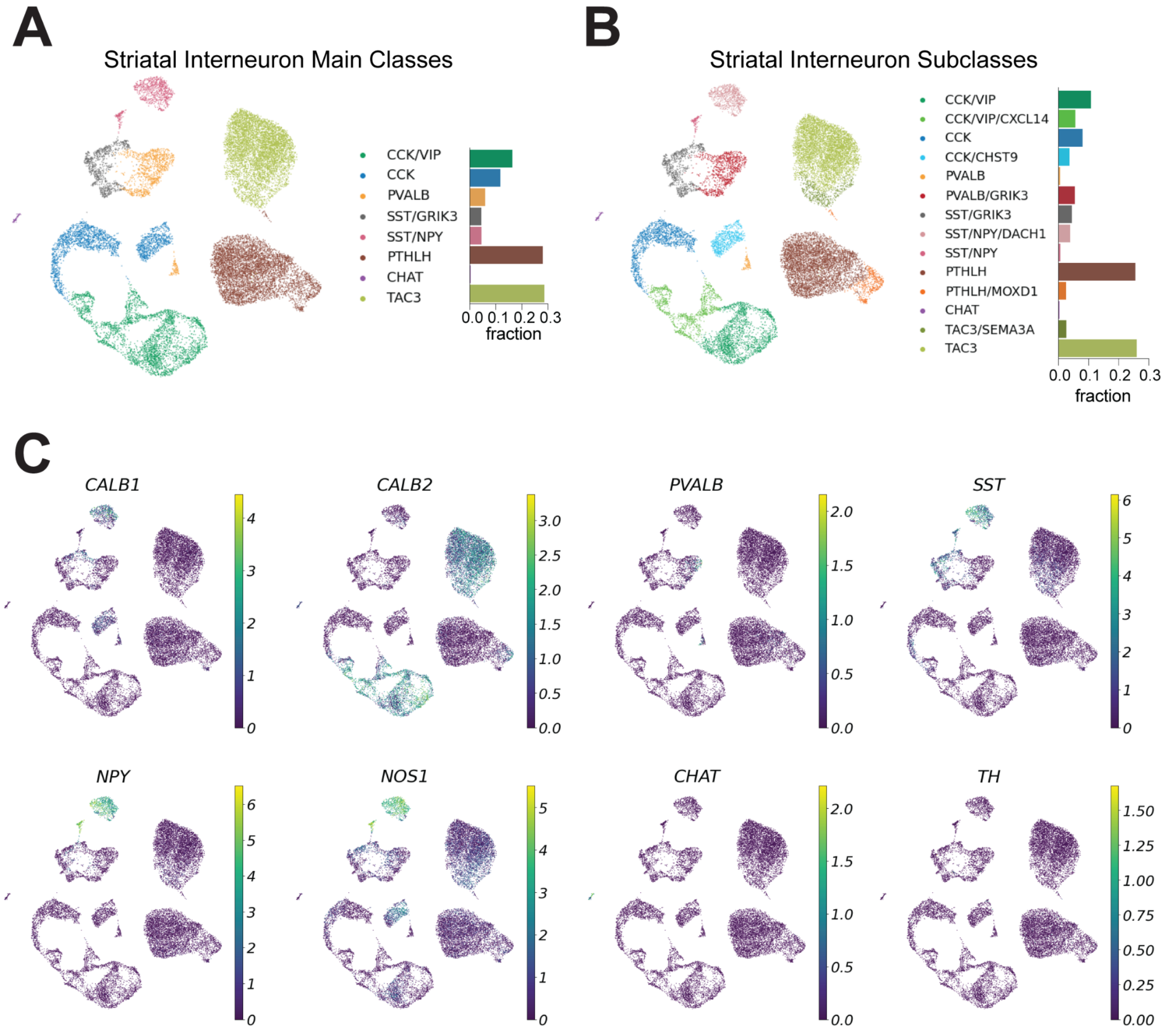

Supplementary Figure 3. Interneuron classes and subclasses and expression of classical markers. A. (left) uniform manifold approximation projection (UMAP) of the interneuron nuclei colored by main class. (right) The barplot indicates the fraction of each class over all interneuron nuclei. B. (left) UMAP of the interneuron nuclei colored by subclass. (right) The barplot indicates the fraction of each subclass over all interneuron nuclei. C. UMAP of the interneuron nuclei colored by the normalized expression of different classical interneuron markers.

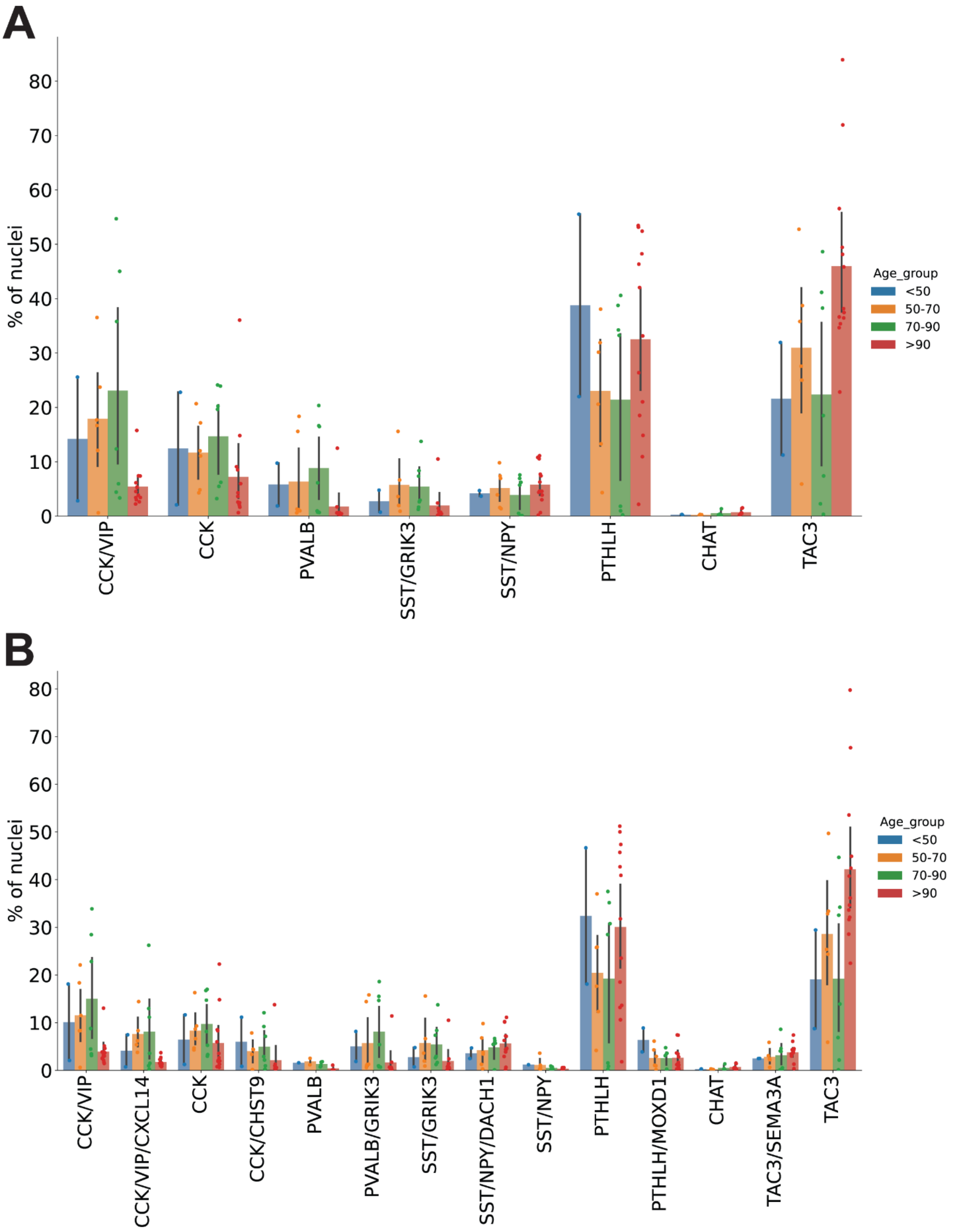
A
% of nuclei
0
10
20
30
40
50
60
70
80
CCK/VIP
CCK
PVALB
SST/GRIK3
SST/NPY
PTHLH
CHAT
TAC3
Age_group
<50
50-70
70-90
>90
B
% of nuclei
0
10
20
30
40
50
60
70
80
CCK/VIP
CCK/VIP/CXCL14
CCK
CCK/CHST9
PVALB
PVALB/GRIK3
SST/GRIK3
SST/NPY/DACH1
SST/NPY
PTHLH
PTHLH/MOXD1
CHAT
TAC3/SEMA3A
TAC3
Age_group
<50
50-70
70-90
>90

Supplementary Figure 4. Distribution of interneuron classes and subclasses across different age groups. A. Proportion of nuclei labeled as interneurons assigned to each of the seven interneuron classes across the different age groups. The number of donors per group are as follows: <50 years – 2 donors, 50-70 years – 6 donors, 70-90 years – 7 donors and >90 years – 13 donors. The error bars indicate the 95% confidence interval of the mean of each group. B. Proportion of nuclei labeled as interneurons assigned to each of the 14 interneuron subclasses across the different age groups. The error bars indicate the 95% confidence interval of the mean of each group.

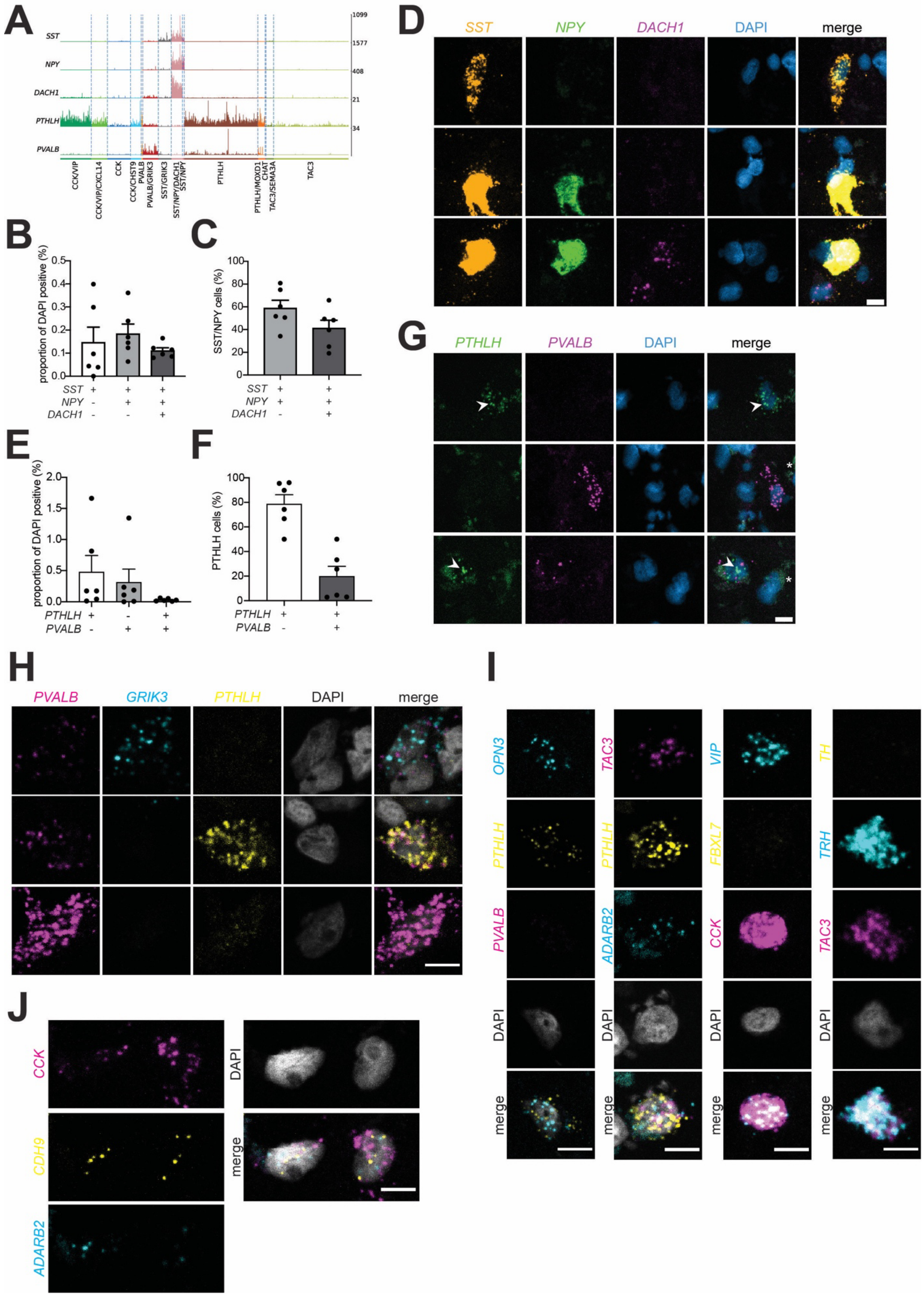
A
SST
NPY
DACH1
PTHLH
PVALB
1099
1577
408
21
34
CCK/VIP
CCK/VIP/CXCL14
CCK
CCK/CHST9
PVALB
PVALB/GRIK3
SST/GRIK3
SST/NPY/DACH1
SST/NPY
PTHLH
PTHLH/MOXD1
CHAT
TAC3/SEMA3A
TAC3
B
proportion of DAPI positive (%)
SST
NPY
DACH1
C
SST/NPY cells (%)
D
SST
NPY
DACH1
DAPI
merge
E
proportion of DAPI positive (%)
PTHLH
PVALB
F
PTHLH cells (%)
G
PTHLH
PVALB
DAPI
merge
H
PVALB
GRIK3
PTHLH
DAPI
merge
I
OPN3
TAC3
VIP
TH
PTHLH
PTHLH
FBXL7
TRH
PVALB
ADARB2
CCK
TAC3
DAPI
merge
J
CCK
CDH9
ADARB2
DAPI
merge

Supplementary Figure 5. Validation using quantitative fluorescence *in-situ* hybridization confirms the existence of distinctive interneuron subclasses in the putamen. A. Trackplot depicting raw UMI counts per nucleus for *SST, NPY*, *DACH1, PTHLH*, and *PVALB* in caudate and putamen. B. Proportion of cells positive for *SST*, *SST* and *NPY* or *SST*, *NPY*, and *DACH1* based on the total number of cells identified per donor (N = 6, putamen). C. Proportion of *SST* and *NPY* double-positive cells negative or positive for *DACH1* (N = 6, putamen). D. Representative images for (top) single-, (middle) double-, and (bottom) triple-positive cells from the same donor, scale bar 10 µm. E. Proportion of positive cells for *PTHLH*, *PVALB*, or *PTHLH* and *PVALB* based on the total number of cells identified per donor (N = 6, putamen). F. Proportion of *PTHLH* positive cells negative or positive for *PVALB* (N = 6, putamen). G. Representative images for (top and middle) single- or (bottom) double-positive cells from the same donor. White arrows indicate spot signal for *PTHLH*, whereas asterisks mark autofluorescence due to lipofuscin, scale bar 10 µm. H. Single channel image panel for representative cells of the *PVALB*, *GRIK3*, and *PTHLH* staining, all from the same donor, scale bar 10 µm. I. Single channel image column for (first) *OPN3*, *PTHLH*, and *PVALB* staining, (second) *TAC3*, *PTHLH*, *ADARB2* staining, (third) *VIP*, *FBXL7*, *CCK* staining and (last) *TH*, *TRH*, and *TAC3* staining, scale bar 10 µm. J. Single channel image panel for representative cells of the *CCK*, *CDH9*, and *ADARB2* staining, scale bar 10 µm. Bigger versions of the merge images of all stainings in Figure 3F. All error bars are depicted as SEM.

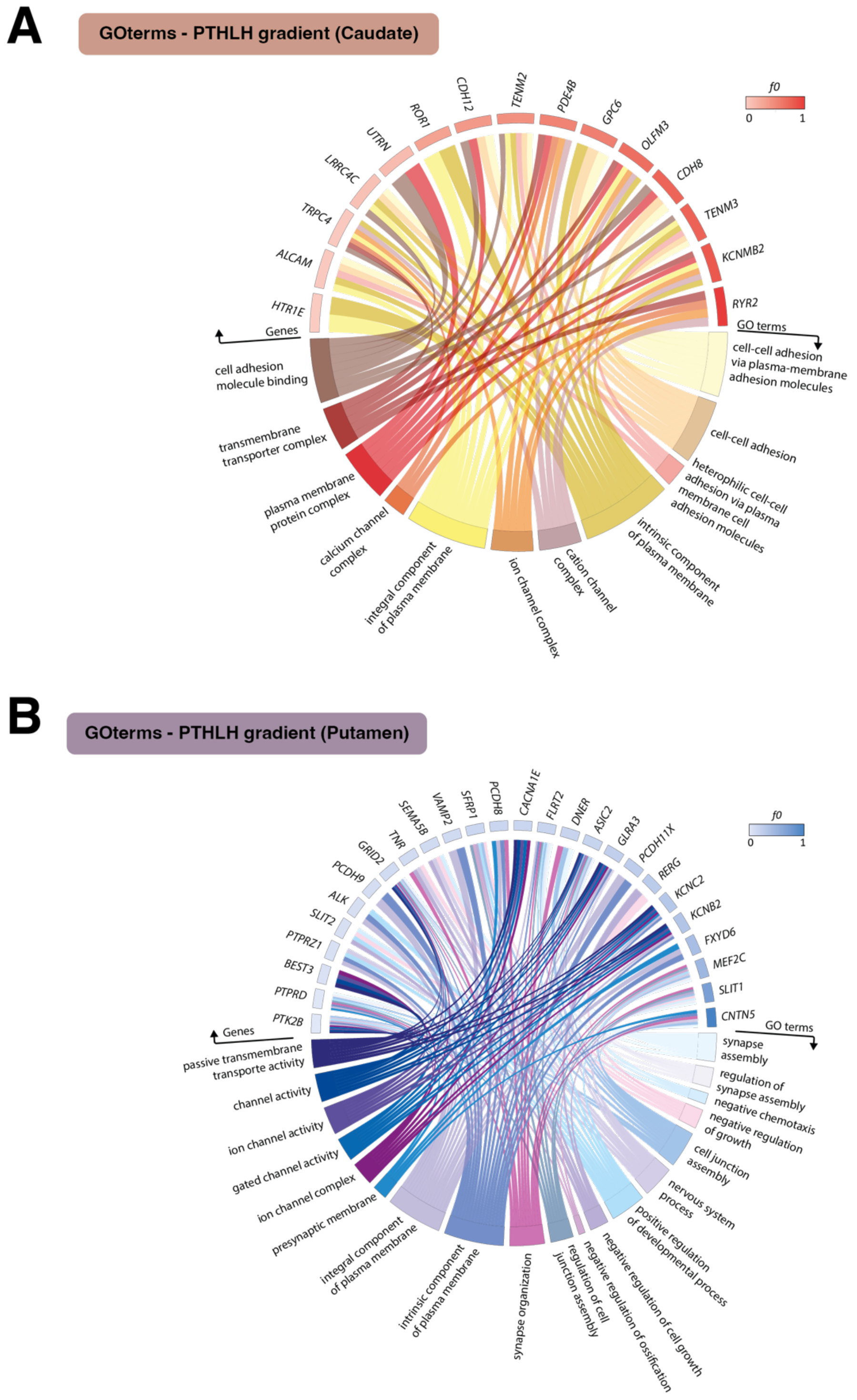

Supplementary Figure 6. GO enrichment analysis of genes correlated positively with the PTHLH vector from the factor analysis. A. GO circos plot representing enriched terms (adjusted p-value < 0.05) with their respective enriched genes along with the f0 value of these genes. Genes correlated positively with the vector in PTHLH subclass in caudate nucleus (f0 > 0.2) were selected. B. Same as A but with genes correlated positively with the vector in PTHLH subclass in putamen. Both sets of genes show similar GO-terms enrichment.

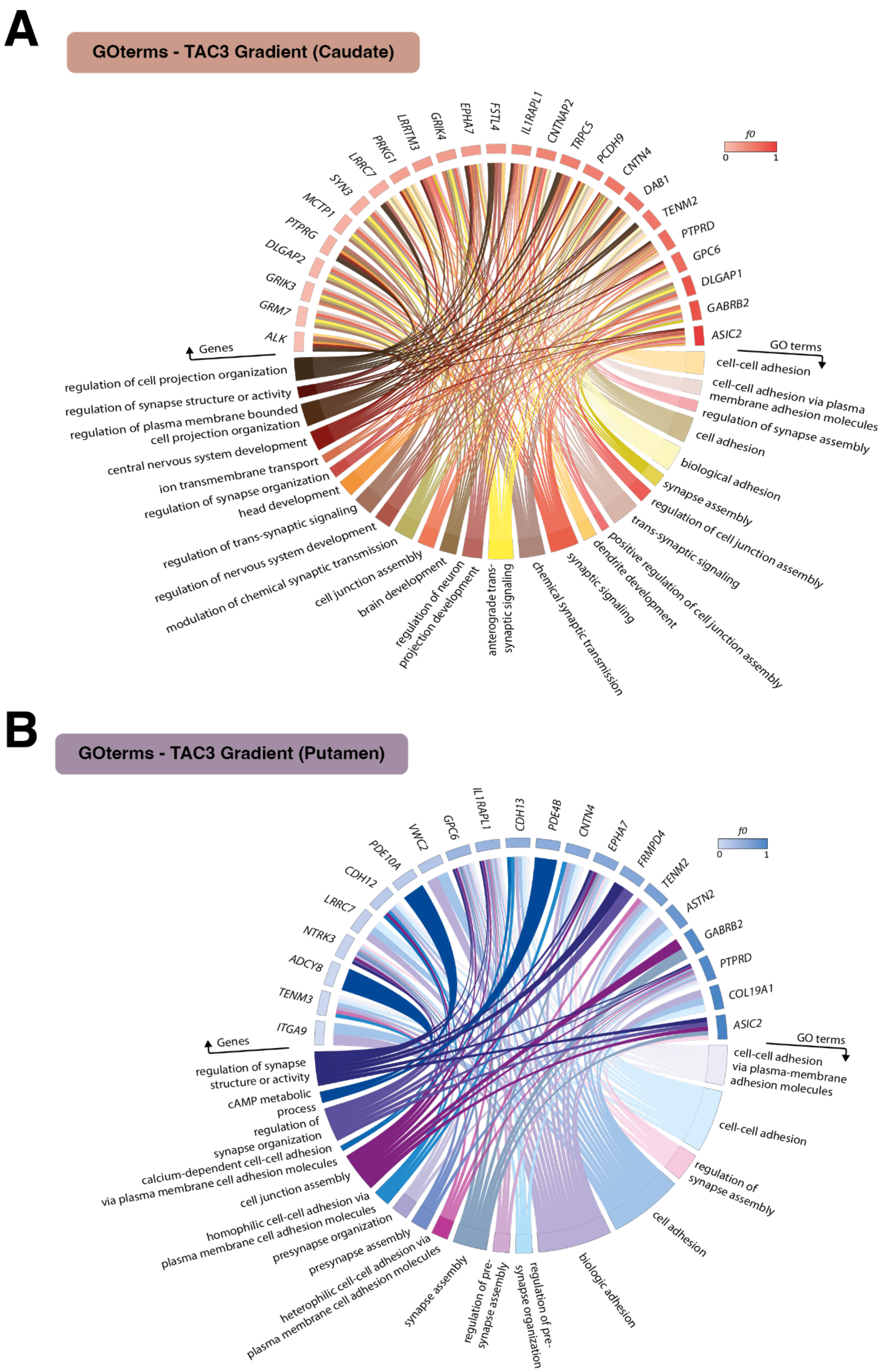

Supplementary Figure 7. GO enrichment analysis of genes correlated positively with the TAC3 vector from the factor analysis. A. GO circos plot representing enriched terms (adjusted p-value < 0.05) with their respective enriched genes along with the f0 value of these genes. Genes correlated positively with the vector in TAC3 subclass in caudate (f0 > 0.2) were selected. B. Same as A but with genes correlated positively with the vector in TAC3 subclass in putamen. Both sets of genes show similar GO-terms enrichment.

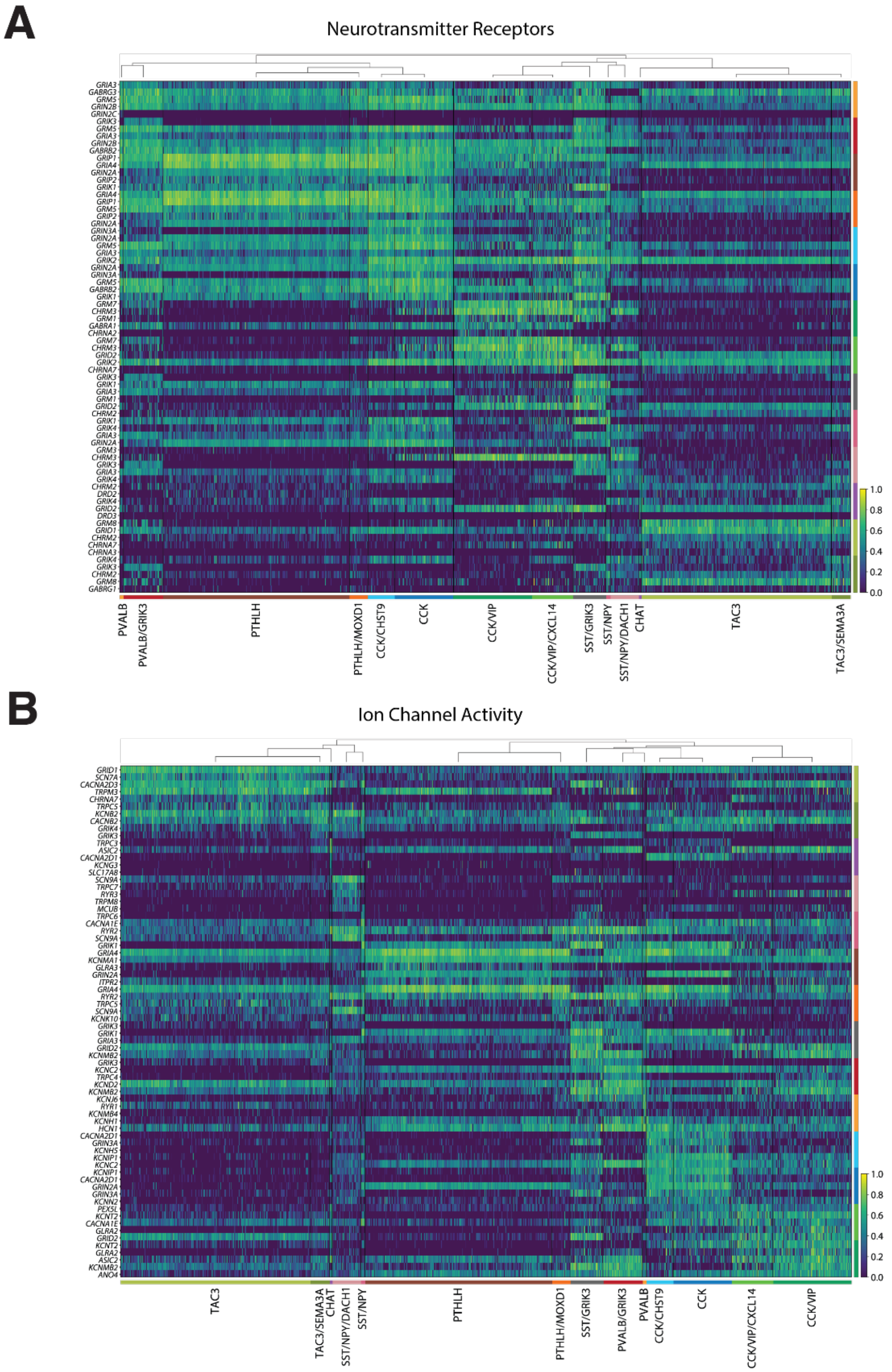

Supplementary Figure 8. Differences in expression of genes encoding neurotransmitter receptors and ion channels across human striatal interneuron subclasses. A. Heatmap depicting the top five differentially expressed genes by interneuron subclass among the neurotransmitter receptor genes. The dendrogram on top of the plot was computed based on the average Pearson correlation coefficient between groups across the selected subset of genes. B. Heatmap including the top five differentially expressed genes by subclass in the ion channel subset. The dendrogram is computed as in A but considering only the subset of genes with ion channel activity in this case.

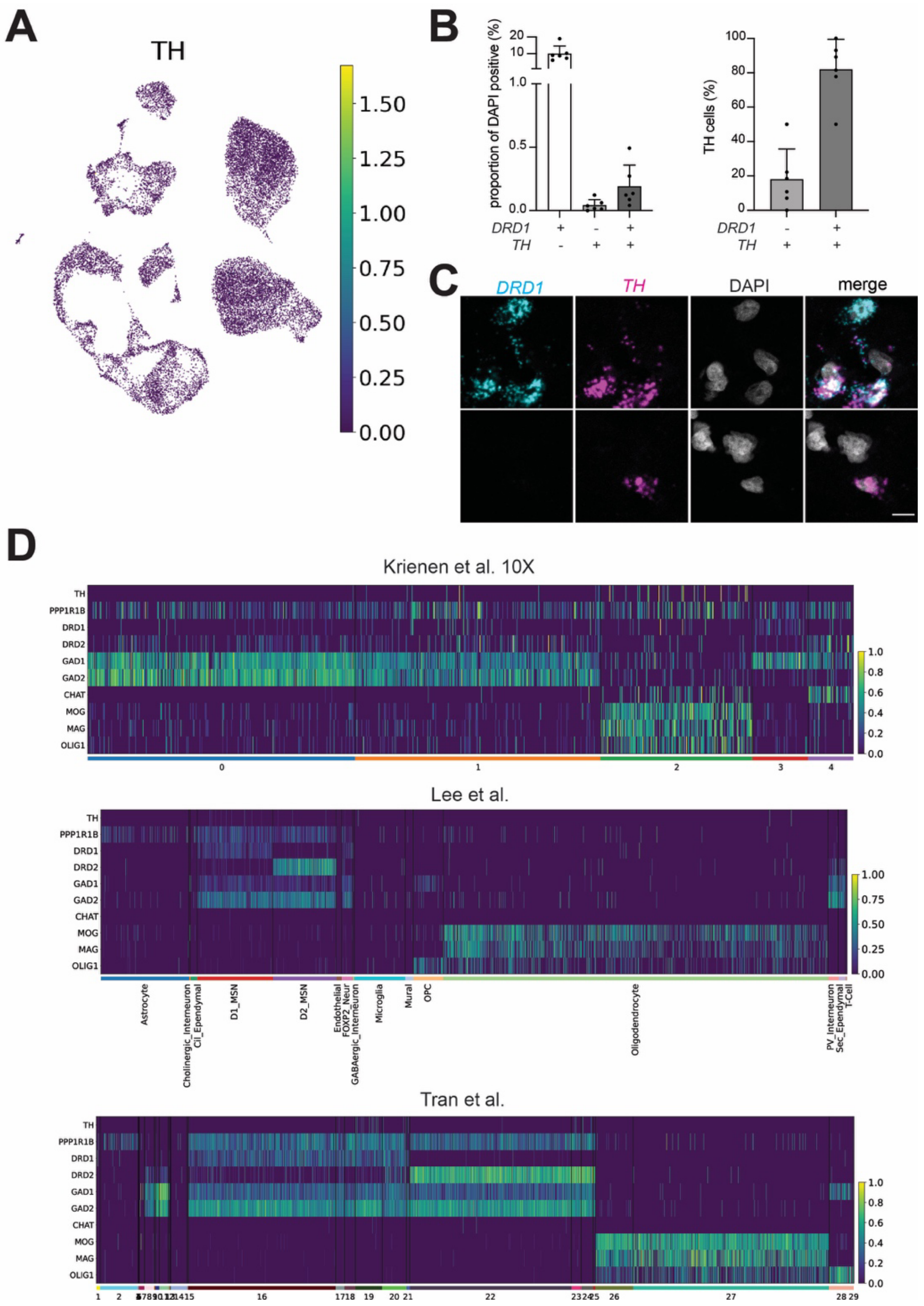

Supplementary Figure 9. Expression of *TH* in human striatal interneurons. A. Uniform manifold approximation projection (UMAP) of the snRNA-seq data of the nuclei labeled as interneurons colored by the level of normalized *TH* expression. B. (left) Proportion of cells positive for *DRD1*, *TH*, or *DRD1* and *TH* based on the total number of cells identified per donor (N = 6, putamen). (right) Proportion of *TH* positive cells negative or positive for *DRD1* (N = 6, putamen). C. Representative images for (top) double- and (bottom) single-positive cells from the same donor, scale bar 10 µm. D. Expression of *TH*, MSN markers (*PPP1R1B*, *DRD1*, *DRD2*), Interneuron markers (*GAD1*, *GAD2*, *CHAT*) and oligodendrocyte markers in the datasets from Krienen *et al*[35]., Lee *et al*.[37] and Tran *et al*.[91] *TH* was not present in the DropSeq dataset from the study by Krienen *et al*. and therefore it was not included here. The cells are either grouped by the labels provided by the authors (Lee *et al*.) or by a cluster number obtained after applying dimensionality reduction and the Louvain algorithm. All error bars are depicted as SD.

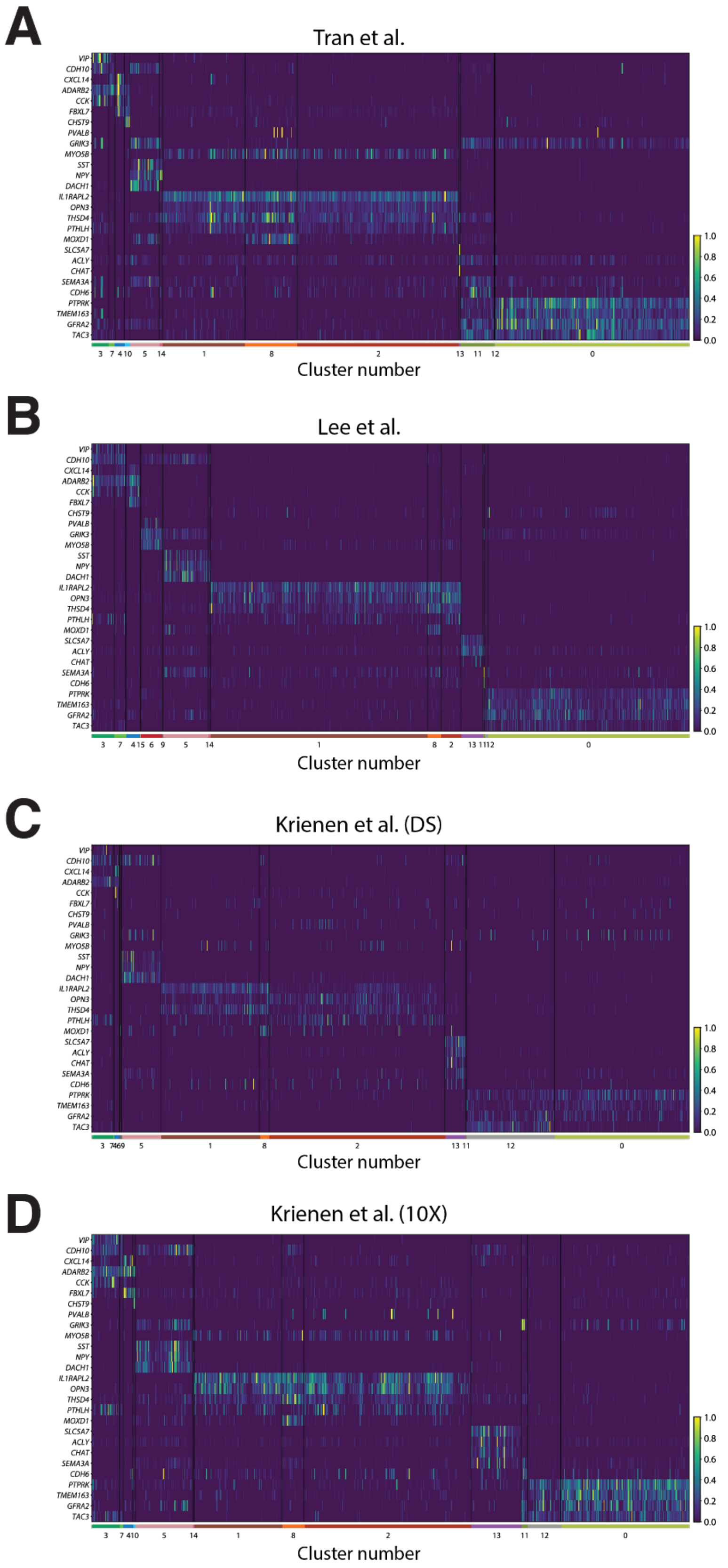

Supplementary Fig. 10. Expression of interneuron subclass markers on the raw counts of each of the public datasets used in the study. Each panel is labeled after the origin of the data. The raw counts of each gene are normalized by the maximum value of counts for that gene within each dataset (i.e. per row). The cluster numbers correspond to those on Figure 6, and the expression patterns follow the relationship established with the interneuron subclasses.

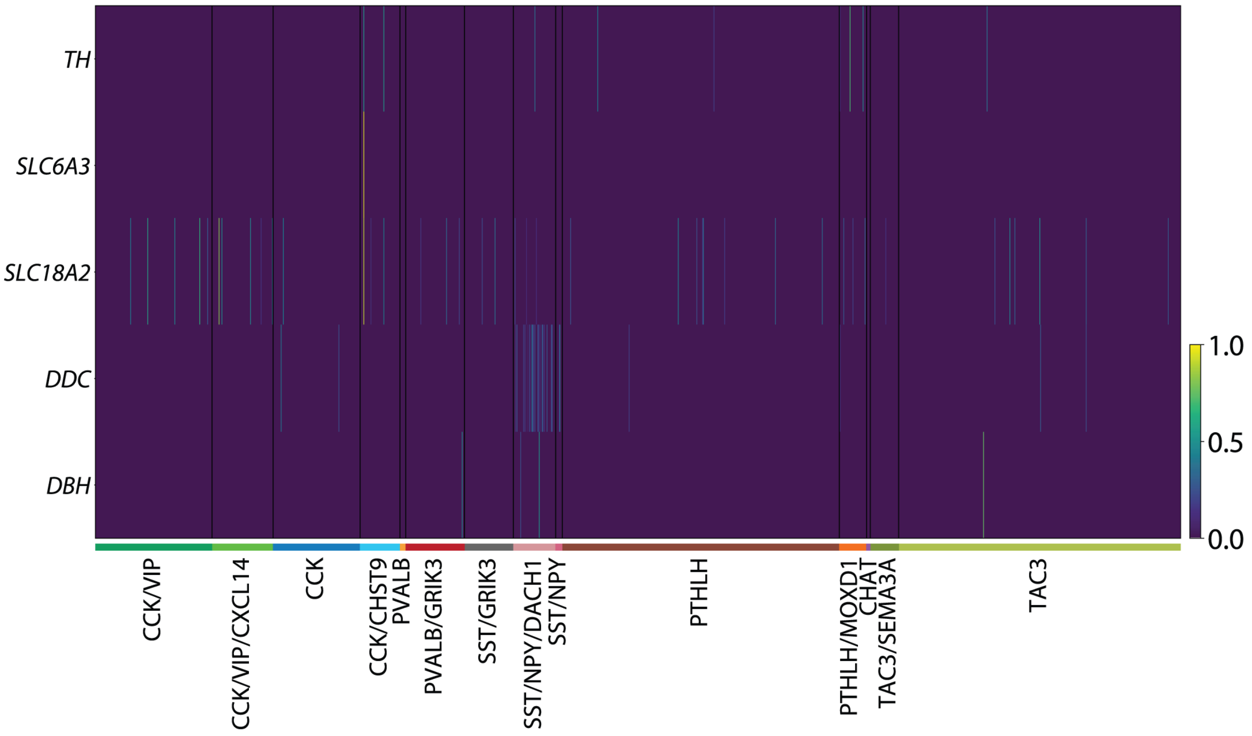

Supplementary Fig. 11. Expression of dopaminergic machinery genes across the interneuron subclasses. The panel shows the normalized expression of tyrosine hydroxylase (*TH*), dopamine transporter (*SLC6A3*), Vesicular Monoamine Transporter-2 (*SLC18A2*), aromatic amino acid decarboxylase (*DDC*) and dopamine beta hydroxylase (DBH) genes.
