# Supplementary material for: Interneuron diversity in the human dorsal striatum
Source: Nat Commun. 2024 Jul 22;15:6164. doi: 10.1038/s41467-024-50414-w (PMC11263574; doi:10.1038/s41467-024-50414-w)
Supplement: Supplementary file 3 — Description of Additional Supplementary Files [file 41467_2024_50414_MOESM3_ESM.pdf]

Supplementary Data Legends

Supplementary Dataset 1. Specimen information**.** Overview on human sample including information about origin, age, brain region and sequencing information. MADRC, Massachusetts Alzheimer's Disease Research Center (Charlstown, USA); NIH, Human Brain and Spinal Fluid Resource Center (Los Angeles, USA); PUKBB, Parkinson's UK Brain Bank at Imperial (London, UK); PMI, post-mortem interval.

Supplementary Dataset 2. Differential gene expression by interneuron class and subclass. Analysis performed at the cell level using a Wilcoxon rank-sum test with Benjamini-Hochberg p-value correction.

Supplementary Dataset 3. Differential gene expression by region. The analysis was conducted using a pseudo-bulk approach, aggregating cells by sample.

Supplementary Dataset 4. Weights assigned to genes on the factor analysis of the TAC3 and PTHLH subclasses.

Supplementary Dataset 5. Differential gene expression by interneuron class and subclass among neurotransmitter receptor genes and genes with ion channel activity. Analysis performed at the cell level using a Wilcoxon rank-sum test with Benjamini-Hochberg p-value correction.

Supplementary Dataset 6. Description of public datasets, number of nuclei filtered at each step of the analysis and number of nuclei per dataset.

Supplementary Dataset 7. QuPath thresholds and cutoffs used in the validation experiments.

Supplementary Dataset 8. Abbreviations used in the text.
