# Supplementary material for: Interneuron diversity in the human dorsal striatum
Source: Nat Commun. 2024 Jul 22;15:6164. doi: 10.1038/s41467-024-50414-w (PMC11263574; doi:10.1038/s41467-024-50414-w)
Supplement: Supplementary file 5 — Reporting Summary [file 41467_2024_50414_MOESM5_ESM.pdf]

nature portfolio

Corresponding author(s): Ana B. Muñoz-Manchado

Last updated by author(s): 2024/06/21

Reporting Summary

Nature Portfolio wishes to improve the reproducibility of the work that we publish. This form provides structure for consistency and transparency in reporting. For further information on Nature Portfolio policies, see our Editorial Policies and the Editorial Policy Checklist.

Statistics

For all statistical analyses, confirm that the following items are present in the figure legend, table legend, main text, or Methods section.

n/a	Confirmed	
☐	☒	The exact sample size (*n*) for each experimental group/condition, given as a discrete number and unit of measurement
☐	☒	A statement on whether measurements were taken from distinct samples or whether the same sample was measured repeatedly
☐	☒	The statistical test(s) used AND whether they are one- or two-sided<br>*Only common tests should be described solely by name; describe more complex techniques in the Methods section.*
☒	☐	A description of all covariates tested
☐	☒	A description of any assumptions or corrections, such as tests of normality and adjustment for multiple comparisons
☒	☐	A full description of the statistical parameters including central tendency (e.g. means) or other basic estimates (e.g. regression coefficient) AND variation (e.g. standard deviation) or associated estimates of uncertainty (e.g. confidence intervals)
☐	☒	For null hypothesis testing, the test statistic (e.g. *F*, *t*, *r*) with confidence intervals, effect sizes, degrees of freedom and *P* value noted<br>*Give P values as exact values whenever suitable.*
☒	☐	For Bayesian analysis, information on the choice of priors and Markov chain Monte Carlo settings
☒	☐	For hierarchical and complex designs, identification of the appropriate level for tests and full reporting of outcomes
☒	☐	Estimates of effect sizes (e.g. Cohen's *d*, Pearson's *r*), indicating how they were calculated

Our web collection on statistics for biologists contains articles on many of the points above.

Software and code

Policy information about availability of computer code

Data collection	FACSDiva V8.0.2 and V9.0.1, Zen 2.6 (Zeiss)
Data analysis	CellRanger v3.0.0 (10X Genomics), Python 3.9.7, Scanpy 1.9.1, Libra 1.0.0, Scikit-learn 1.5.3, clusterProfiler 3.16,<br>QuPath 0.3.2, scVI 0.8.1<br>No custom algorithms or software was used. The software and libraries used for the analysis is referenced in the manuscript.

For manuscripts utilizing custom algorithms or software that are central to the research but not yet described in published literature, software must be made available to editors and reviewers. We strongly encourage code deposition in a community repository (e.g. GitHub). See the Nature Portfolio guidelines for submitting code & software for further information.

Data

Policy information about availability of data

All manuscripts must include a data availability statement. This statement should provide the following information, where applicable:

- Accession codes, unique identifiers, or web links for publicly available datasets
- A description of any restrictions on data availability
- For clinical datasets or third party data, please ensure that the statement adheres to our policy

Single-cell RNA-seq data have been deposited at Figshare and are publicly available as of the date of publication (doi:10.6084/m9.figshare.22340140).
Xenium in situ data has been deposited at Figshare and Zenodo and is available as of the date of publication (Figshare doi: 10.6084/m9.figshare.25975132; Zenodo

doi: 10.5281/zenodo.11609973; doi: 10.5281/zenodo; 11534381; doi: 10.5281/zenodo.11612060).

Research involving human participants, their data, or biological material

Policy information about studies with human participants or human data. See also policy information about sex, gender (identity/presentation), and sexual orientation and race, ethnicity and racism.

Reporting on sex and gender	We have reported the number of human donors of each sex. We do not have information about their gender, thus none is reported. The raw data contain sex information.
Reporting on race, ethnicity, or other socially relevant groupings	We have no information about the race, ethnicity or other socially relevant groupings of the donors.
Population characteristics	We have reported the age range of the donors from each of the three biobanks involved in the study. Specific age is specified in raw data.
Recruitment	We requested samples from donors that did not meet the diagnostic criteria for any neurodegenerative disease
Ethics oversight	Swedish Ethical Review Authority (Etikprövningsmyndigheten) ref. 2020-00341. Donors or their next-of-kin provided written informed consent for brain autopsy, and the study was approved by the review board of each brain bank.

Note that full information on the approval of the study protocol must also be provided in the manuscript.

Field-specific reporting

Please select the one below that is the best fit for your research. If you are not sure, read the appropriate sections before making your selection.

☒ Life sciences ☐ Behavioural & social sciences ☐ Ecological, evolutionary & environmental sciences

For a reference copy of the document with all sections, see nature.com/documents/nr-reporting-summary-flat.pdf

Life sciences study design

All studies must disclose on these points even when the disclosure is negative.

Sample size	We obtained samples from 28 donors. The sample size was limited by the availability of fresh-frozen striatum samples and the scope of the project, yet we produced the largest snRNA-seq dataset of human striatal interneurons to date.
Data exclusions	None of the samples were excluded from the study.
Replication	To verify the reproducibility of our findings, we reproduced our taxonomy using data from 4 datasets belonging to 3 previous studies. Besides the commonalities found after integrating the data, we show that the expression patterns which define the groups in our taxonomy can be observed in the raw, non-normalized data from published datasets.
Randomization	Our study did not use experimental groups, therefore the samples were not randomized.
Blinding	Our study did not use experimental groups, therefore blinding was not considered necessary.

Behavioural & social sciences study design

All studies must disclose on these points even when the disclosure is negative.

Study description	N/A
Research sample	N/A
Sampling strategy	N/A
Data collection	N/A
Timing	N/A
Data exclusions	N/A
Non-participation	N/A

Randomization	N/A

Ecological, evolutionary & environmental sciences study design

All studies must disclose on these points even when the disclosure is negative.

Study description	N/A
Research sample	N/A
Sampling strategy	N/A
Data collection	N/A
Timing and spatial scale	N/A
Data exclusions	N/A
Reproducibility	N/A
Randomization	N/A
Blinding	N/A

Did the study involve field work? ☐ Yes ☒ No

Field work, collection and transport

Field conditions	N/A
Location	N/A
Access & import/export	N/A
Disturbance	N/A

Reporting for specific materials, systems and methods

We require information from authors about some types of materials, experimental systems and methods used in many studies. Here, indicate whether each material, system or method listed is relevant to your study. If you are not sure if a list item applies to your research, read the appropriate section before selecting a response.

Materials & experimental systems

n/a	Involved in the study	
☐	☒	Antibodies
☒	☐	Eukaryotic cell lines
☒	☐	Palaeontology and archaeology
☒	☐	Animals and other organisms
☒	☐	Clinical data
☒	☐	Dual use research of concern
☒	☐	Plants

Methods

n/a	Involved in the study	
☒	☐	ChIP-seq
☐	☒	Flow cytometry
☒	☐	MRI-based neuroimaging

Antibodies

Antibodies used	Millimark Mouse Anti-NeuN PE Conjugated, Clone A60 (Merck, identifier FCMAB317PE)
Validation	The antibody was validated by the supplier for use in flow cytometry for the detection of NeuN, using U251 cells.<br>All further information can be found on the suppliers webpage:<br>https://www.merckmillipore.com/SE/en/product/Milli-Mark-Anti-NeuN-PE-Antibody-clone-A60,MM_NF-FCMAB317PE

Eukaryotic cell lines

Policy information about cell lines and Sex and Gender in Research

Cell line source(s)	N/A
Authentication	N/A
Mycoplasma contamination	N/A
Commonly misidentified lines (See ICLAC register)	N/A

Palaeontology and Archaeology

Specimen provenance	N/A
Specimen deposition	N/A
Dating methods	N/A

☐ Tick this box to confirm that the raw and calibrated dates are available in the paper or in Supplementary Information.

Ethics oversight	N/A

Note that full information on the approval of the study protocol must also be provided in the manuscript.

Animals and other research organisms

Policy information about studies involving animals; ARRIVE guidelines recommended for reporting animal research, and Sex and Gender in Research

Laboratory animals	N/A
Wild animals	N/A
Reporting on sex	N/A
Field-collected samples	N/A
Ethics oversight	N/A

Note that full information on the approval of the study protocol must also be provided in the manuscript.

Clinical data

Policy information about clinical studies

All manuscripts should comply with the ICMJE guidelines for publication of clinical research and a completed CONSORT checklist must be included with all submissions.

Clinical trial registration	N/A
Study protocol	N/A
Data collection	N/A
Outcomes	N/A

Dual use research of concern

Policy information about dual use research of concern

Hazards

Could the accidental, deliberate or reckless misuse of agents or technologies generated in the work, or the application of information presented in the manuscript, pose a threat to:

No	Yes	
☐	☐	Public health
☐	☐	National security
☐	☐	Crops and/or livestock
☐	☐	Ecosystems
☐	☐	Any other significant area

Experiments of concern

Does the work involve any of these experiments of concern:

No	Yes	
☐	☐	Demonstrate how to render a vaccine ineffective
☐	☐	Confer resistance to therapeutically useful antibiotics or antiviral agents
☐	☐	Enhance the virulence of a pathogen or render a nonpathogen virulent
☐	☐	Increase transmissibility of a pathogen
☐	☐	Alter the host range of a pathogen
☐	☐	Enable evasion of diagnostic/detection modalities
☐	☐	Enable the weaponization of a biological agent or toxin
☐	☐	Any other potentially harmful combination of experiments and agents

Plants

Seed stocks	n/a
Novel plant genotypes	n/a
Authentication	n/a

ChIP-seq

Data deposition

☐ Confirm that both raw and final processed data have been deposited in a public database such as GEO.

☐ Confirm that you have deposited or provided access to graph files (e.g. BED files) for the called peaks.

Data access links<br>*May remain private before publication.*	N/A
Files in database submission	N/A
Genome browser session<br>(e.g. UCSC)	N/A

Methodology

Replicates	N/A
Sequencing depth	N/A
Antibodies	N/A
Peak calling parameters	N/A
Data quality	N/A
Software	N/A

Flow Cytometry

Plots

Confirm that:

☒ The axis labels state the marker and fluorochrome used (e.g. CD4-FITC).

☒ The axis scales are clearly visible. Include numbers along axes only for bottom left plot of group (a 'group' is an analysis of identical markers).

☒ All plots are contour plots with outliers or pseudocolor plots.

☒ A numerical value for number of cells or percentage (with statistics) is provided.

Methodology

Sample preparation	Nuclei were isolated from 100 – 150 mg of fresh frozen tissue using nuclease-free homogenization buffer (10 mM Tris (pH 8), 250 mM Sucrose, 25 mM KCl, 5 mM MgCl2, 0.1 mM DTT, 1x Protease inhibitor cocktail (50x in 100 % Ethanol, G6521, Promega), 0.2 U/µl RNasin Plus (N2615, Promega), 0.1 % Triton X-100) and a dounce tissue grinder with loose and tight pestle (20 strokes each, 357538, Wheaton). The nuclei were separated from debris using a Iodixanol gradient (OptiPrep Density Gradient Medium (D1556, Sigma) in 60 mM Tris (pH 8), 250 mM Sucrose, 150 mM KCl, 30 mM MgCl2) and incubated with NeuN antibody for 30 minutes (1:500, Millimark mouse anti-NeuN PE conjugated (FCMAB317PE, Merck) in nuclease-free blocking buffer (1x PBS, 1 % BSA, 0.2 U/µl RNasin Plus)). After removing the antibody, nuclei were resuspended in 500 µl blocking buffer and stained with DAPI (final concentration 0.05 mg/ml, (D3571, Invitrogen)).
Instrument	BD FACSAria Fusion Flow Cytometer<br>BD FACSAria III Cell Sorter
Software	FACSDiva V8.0.2 and V9.0.1<br>FlowJo 10.8.2
Cell population abundance	Samples were sorted until collecting 200000 NeuN positive and 200000 NeuN negative nuclei or until the entire sample was used.
Gating strategy	FSC-H versus DAPI-A was used to distinguish nuclei from debris, followed by FSC-H versus FSC-A to separate single nuclei from doublets. Finally PE-A versus DAPI-A was used to identify NeuN positive and NeuN negative nuclei and sort each fraction in a separate tube.

☐ Tick this box to confirm that a figure exemplifying the gating strategy is provided in the Supplementary Information.

Magnetic resonance imaging

Experimental design

Design type	N/A
Design specifications	N/A
Behavioral performance measures	N/A

Acquisition

Imaging type(s)	N/A
Field strength	N/A
Sequence & imaging parameters	N/A
Area of acquisition	N/A

Diffusion MRI ☐ Used ☒ Not used

Preprocessing

Preprocessing software	N/A
Normalization	N/A
Normalization template	N/A

Noise and artifact removal	N/A
Volume censoring	N/A

Statistical modeling & inference

Model type and settings	N/A
Effect(s) tested	N/A

Specify type of analysis: ☐ Whole brain ☐ ROI-based ☐ Both

Statistic type for inference (See Eklund et al. 2016)	N/A
Correction	N/A

Models & analysis

n/a	Involved in the study
☐	☐ Functional and/or effective connectivity
☐	☐ Graph analysis
☐	☐ Multivariate modeling or predictive analysis

Functional and/or effective connectivity	N/A
Graph analysis	N/A
Multivariate modeling and predictive analysis	N/A
